# Supplementary material for: Rethinking model prototyping through the MedMNIST+ dataset collection
Source: Sci Rep. 2025 Mar 5;15:7669. doi: 10.1038/s41598-025-92156-9 (PMC11883007; doi:10.1038/s41598-025-92156-9)
Supplement: Supplementary file 1 — Supplementary Information. [file 41598_2025_92156_MOESM1_ESM.pdf]

## Appendix

**Supplementary Table S1.** Benchmark outcomes summarizing the mean and standard deviation of accuracy (ACC), for a fixed operating point of 0.5, and area under the receiver operating characteristic curve (AUC) for the BloodMNIST dataset across all training scheme-model-image resolution combinations, derived from three independent random seeds. Notably, the $k$-NN algorithm, devoid of a training phase, remains unaffected by the stochasticity inherent in model training, thus reporting only the total ACC value without standard deviation. Moreover, owing to its direct utilization of embeddings and labels for classification, $k$-NN does not furnish a reliable AUC score. The overall best result across all training schemes, models, and resolutions is highlighted with a background color ; the best result per resolution across all training schemes and models is highlighted with underline; and the best result per training scheme and resolution is highlighted in **bold**.

| **BloodMNIST** | | | | | | | | |
|---|---|---|---|---|---|---|---|---|
| Methods | Accuracy (ACC) | | | | Area Under the ROC Curve (AUC) | | | |
| | 28 × 28 | 64 × 64 | 128 × 128 | 224 × 224 | 28 × 28 | 64 × 64 | 128 × 128 | 224 × 224 |
| END-TO-END | | | | | | | | |
| VGG16 | **94.85±0.47** | **98.20±0.16** | 98.52±0.25 | 98.77±0.31 | **99.66±0.06** | **99.91±0.00** | **99.93±0.01** | **99.95±0.01** |
| AlexNet | 90.39±0.46 | 96.41±0.50 | 97.74±0.29 | 98.18±0.39 | 99.12±0.04 | 99.83±0.00 | 99.89±0.01 | 99.92±0.00 |
| ResNet-18 | 91.93±0.37 | 97.06±0.11 | 98.34±0.26 | 98.94±0.08 | 99.24±0.04 | 99.82±0.01 | 99.92±0.01 | 99.92±0.01 |
| DenseNet-121 | 93.87±0.40 | 97.99±0.28 | **98.90±0.09** | **99.02±0.14** | 99.58±0.03 | **99.91±0.02** | 99.92±0.01 | **99.95±0.01** |
| EfficientNet-B4 | 78.06±1.34 | 90.61±0.39 | 96.00±0.19 | 97.40±0.27 | 96.62±0.26 | 99.26±0.05 | 99.78±0.03 | 99.90±0.00 |
| ViT-B/16 | 90.59±0.56 | 96.42±0.26 | 97.54±0.21 | 98.43±0.09 | 99.21±0.08 | 99.80±0.00 | 99.91±0.01 | 99.93±0.01 |
| CLIP ViT-B/16 | 89.92±0.25 | 93.56±0.90 | 95.60±0.42 | 96.82±0.04 | 99.08±0.03 | 99.56±0.04 | 99.78±0.03 | 99.87±0.01 |
| EVA-02 ViT-B/16 | 91.24±0.63 | 95.65±0.26 | 97.94±0.59 | 98.42±0.12 | 99.15±0.10 | 99.75±0.03 | 99.92±0.02 | 99.93±0.01 |
| DINO ViT-B/16 | 89.87±0.47 | 95.69±0.12 | 97.54±0.54 | 97.98±0.46 | 99.13±0.09 | 99.78±0.01 | 99.89±0.03 | 99.93±0.01 |
| SAM ViT-B/16 | 91.19±0.54 | 95.89±0.12 | 97.33±0.32 | 98.55±0.26 | 99.16±0.07 | 99.72±0.01 | 99.86±0.04 | 99.92±0.02 |
| LINEAR PROBING | | | | | | | | |
| VGG16 | 74.95±0.02 | 84.53±0.04 | 88.86±0.09 | 93.77±0.09 | 95.66±0.00 | 98.24±0.00 | 99.10±0.00 | 99.64±0.00 |
| AlexNet | 66.67±0.25 | 81.97±0.22 | 89.61±0.12 | 91.51±0.02 | 93.84±0.01 | 97.69±0.05 | 99.16±0.02 | 99.39±0.00 |
| ResNet-18 | 63.40±0.13 | 67.15±0.06 | 80.58±0.10 | 91.10±0.10 | 90.58±0.05 | 93.67±0.01 | 97.15±0.00 | 99.25±0.00 |
| DenseNet-121 | 71.34±0.12 | 84.03±0.07 | 93.36±0.02 | 95.65±0.06 | 94.08±0.03 | 97.99±0.01 | 99.55±0.00 | 99.81±0.00 |
| EfficientNet-B4 | 59.46±0.72 | 69.54±0.24 | 84.10±0.17 | 90.19±0.10 | 88.94±0.20 | 93.97±0.06 | 97.91±0.03 | 99.11±0.04 |
| ViT-B/16 | 80.67±0.07 | 93.21±0.12 | 97.07±0.01 | 97.95±0.04 | 97.13±0.01 | 99.50±0.00 | 99.88±0.00 | 99.92±0.00 |
| CLIP ViT-B/16 | 83.05±0.20 | 92.21±0.04 | 96.00±0.02 | 96.13±0.07 | 97.80±0.01 | 99.38±0.01 | 99.77±0.00 | 99.86±0.00 |
| EVA-02 ViT-B/16 | 82.35±0.01 | 89.89±0.00 | 93.10±0.00 | 94.09±0.01 | 97.58±0.00 | 99.03±0.00 | 99.53±0.00 | 99.66±0.00 |
| DINO ViT-B/16 | **88.79±0.04** | **97.15±0.09** | **98.25±0.10** | **98.70±0.01** | **98.81±0.00** | **99.86±0.00** | **99.92±0.00** | **99.94±0.00** |
| SAM ViT-B/16 | 20.00±0.04 | 24.61±0.11 | 34.73±0.17 | 51.70±0.10 | 76.78±0.16 | 81.19±0.18 | 87.54±0.03 | 88.98±0.03 |
| $k$-NN ($k = 11$) | | | | | | | | |
| VGG16 | 71.76 | 75.97 | 79.22 | 81.38 | - | - | - | - |
| AlexNet | 68.34 | 82.23 | 87.55 | 89.94 | - | - | - | - |
| ResNet-18 | 75.04 | 76.18 | 80.27 | 89.04 | - | - | - | - |
| DenseNet-121 | 72.29 | 80.94 | 84.10 | 88.54 | - | - | - | - |
| EfficientNet-B4 | 73.63 | 78.49 | 81.00 | 84.48 | - | - | - | - |
| ViT-B/16 | 70.21 | 78.95 | 92.17 | 94.80 | - | - | - | - |
| CLIP ViT-B/16 | 68.66 | 82.87 | 91.38 | 93.19 | - | - | - | - |
| EVA-02 ViT-B/16 | 77.70 | 84.07 | 89.94 | 92.43 | - | - | - | - |
| DINO ViT-B/16 | **85.24** | **93.04** | **96.67** | **96.81** | - | - | - | - |
| SAM ViT-B/16 | 62.50 | 64.40 | 76.15 | 78.22 | - | - | - | - |

**Supplementary Table S2.** Benchmark outcomes summarizing the mean and standard deviation of accuracy (ACC), for a fixed operating point of 0.5, and area under the receiver operating characteristic curve (AUC) for the BreastMNIST dataset across all training scheme-model-image resolution combinations, derived from three independent random seeds. Notably, the *k*-NN algorithm, devoid of a training phase, remains unaffected by the stochasticity inherent in model training, thus reporting only the total ACC value without standard deviation. Moreover, owing to its direct utilization of embeddings and labels for classification, *k*-NN does not furnish a reliable AUC score. The overall best result across all training schemes, models, and resolutions is highlighted with a background color; the best result per resolution across all training schemes and models is highlighted with underline; and the best result per training scheme and resolution is highlighted in **bold**.

| BreastMNIST | | | | | | | | |
|---|---|---|---|---|---|---|---|---|
| Methods | Accuracy (ACC) | | | | Area Under the ROC Curve (AUC) | | | |
| | 28 × 28 | 64 × 64 | 128 × 128 | 224 × 224 | 28 × 28 | 64 × 64 | 128 × 128 | 224 × 224 |
| END-TO-END | | | | | | | | |
| VGG16 | 85.26±1.05 | **87.82±2.09** | 89.32±0.80 | 88.03±0.80 | 87.48±0.96 | 90.21±0.48 | 92.40±0.68 | 91.11±0.44 |
| AlexNet | **86.54±1.89** | 87.61±1.21 | **90.60±1.09** | **88.46±0.52** | **89.08±2.41** | 89.67±1.38 | **93.97±1.36** | **93.86±0.48** |
| ResNet-18 | 83.97±1.81 | 83.33±0.52 | 85.47±1.51 | 87.18±0.52 | 87.61±1.16 | 87.14±1.77 | 87.78±0.63 | 89.94±0.57 |
| DenseNet-121 | 83.33±2.91 | 85.90±0.91 | 85.68±0.30 | 87.39±0.80 | 86.18±3.25 | **90.56±0.32** | 86.63±0.55 | 89.67±0.57 |
| EfficientNet-B4 | 76.50±2.47 | 74.57±2.88 | 76.50±2.47 | 74.57±1.32 | 75.62±1.69 | 74.09±4.58 | 76.73±2.92 | 70.84±5.18 |
| ViT-B/16 | 82.05±0.52 | 81.62±2.58 | 82.48±0.80 | 83.76±1.09 | 84.83±0.96 | 84.49±4.67 | 83.63±1.49 | 86.18±0.26 |
| CLIP ViT-B/16 | 77.56±3.43 | 76.71±0.60 | 79.06±0.80 | 80.13±1.81 | 75.19±10.6 | 77.58±2.42 | 78.38±0.73 | 77.53±2.64 |
| EVA-02 ViT-B/16 | 74.79±5.50 | 73.08±0.00 | 72.44±1.38 | 82.91±3.86 | 74.88±6.72 | 73.85±1.78 | 78.36±3.35 | 83.08±5.07 |
| DINO ViT-B/16 | 84.83±3.79 | 81.20±0.80 | 79.70±2.36 | 84.40±1.68 | 88.39±2.64 | 81.95±1.58 | 83.58±0.79 | 86.31±2.70 |
| SAM ViT-B/16 | 82.05±0.52 | 77.35±6.04 | 82.91±2.63 | 81.62±2.12 | 80.16±4.38 | 79.16±5.18 | 82.07±7.69 | 78.51±1.13 |
| LINEAR PROBING | | | | | | | | |
| VGG16 | 78.63±0.30 | 77.78±0.60 | 84.62±0.00 | 80.98±0.60 | 83.10±0.24 | 81.86±0.22 | 89.00±0.08 | 85.06±0.17 |
| AlexNet | 77.56±1.05 | 81.62±0.60 | 84.40±0.30 | 86.11±0.80 | 77.39±0.43 | 82.64±0.38 | 90.38±0.35 | **93.47±0.11** |
| ResNet-18 | 73.08±0.00 | 73.08±0.00 | 73.08±0.00 | 73.08±0.00 | 64.77±1.42 | 54.92±1.76 | 61.72±8.75 | 68.33±1.75 |
| DenseNet-121 | 75.00±1.81 | 78.42±0.80 | 78.21±0.91 | 80.77±0.52 | 64.96±10.86 | 73.84±2.94 | 78.43±1.87 | 79.82±0.95 |
| EfficientNet-B4 | 62.18±4.99 | 63.25±5.16 | 61.11±2.18 | 59.62±6.80 | 54.69±5.15 | 52.17±0.82 | 53.73±6.10 | 52.49±2.50 |
| ViT-B/16 | 77.14±0.30 | 80.34±0.60 | 85.04±1.51 | 84.40±1.32 | 78.22±1.36 | 81.53±1.96 | 90.23±0.19 | 91.15±0.71 |
| CLIP ViT-B/16 | 79.27±0.30 | 81.20±1.32 | 85.26±0.91 | 83.55±0.30 | 75.48±0.31 | 84.40±1.65 | 89.73±0.47 | 86.52±0.73 |
| EVA-02 ViT-B/16 | 76.07±0.30 | 78.63±0.30 | 80.98±0.30 | 82.05±0.00 | 76.68±0.04 | 80.95±0.11 | 86.86±0.07 | 85.46±0.03 |
| DINO ViT-B/16 | **83.33±0.52** | **85.68±0.60** | **88.89±0.80** | **88.68±1.09** | **86.77±0.44** | **91.70±0.27** | **93.25±0.89** | 93.43±0.82 |
| SAM ViT-B/16 | 73.08±0.00 | 73.08±0.00 | 73.08±0.00 | 73.08±0.00 | 48.26±2.40 | 61.65±3.48 | 70.64±0.64 | 73.75±0.40 |
| *k*-NN ($k = 11$) | | | | | | | | |
| VGG16 | 74.36 | 80.77 | 80.77 | 79.49 | - | - | - | - |
| AlexNet | 81.41 | 81.41 | 82.69 | 82.69 | - | - | - | - |
| ResNet-18 | 78.21 | 81.41 | **85.90** | 80.77 | - | - | - | - |
| DenseNet-121 | 78.85 | 75.64 | 83.33 | 84.62 | - | - | - | - |
| EfficientNet-B4 | **83.33** | 81.41 | 83.97 | 86.54 | - | - | - | - |
| ViT-B/16 | 75.64 | 79.49 | 83.97 | 81.41 | - | - | - | - |
| CLIP ViT-B/16 | 78.21 | 78.21 | 80.13 | 80.13 | - | - | - | - |
| EVA-02 ViT-B/16 | 75.64 | 82.69 | 82.69 | 81.41 | - | - | - | - |
| DINO ViT-B/16 | 78.21 | **86.54** | 85.26 | **87.18** | - | - | - | - |
| SAM ViT-B/16 | 74.36 | 82.69 | 77.56 | 78.85 | - | - | - | - |

**Supplementary Table S3.** Benchmark outcomes summarizing the mean and standard deviation of accuracy (ACC), for a fixed operating point of 0.5, and area under the receiver operating characteristic curve (AUC) for the ChestMNIST dataset across all training scheme-model-image resolution combinations, derived from three independent random seeds. Notably, the $k$-NN algorithm, devoid of a training phase, remains unaffected by the stochasticity inherent in model training, thus reporting only the total ACC value without standard deviation. Moreover, owing to its direct utilization of embeddings and labels for classification, $k$-NN does not furnish a reliable AUC score. The overall best result across all training schemes, models, and resolutions is highlighted with a background color; the best result per resolution across all training schemes and models is highlighted with underline; and the best result per training scheme and resolution is highlighted in **bold**.

| ChestMNIST | | | | | | | | |
|---|---|---|---|---|---|---|---|---|
| Methods | Accuracy (ACC) | | | | Area Under the ROC Curve (AUC) | | | |
| | 28 × 28 | 64 × 64 | 128 × 128 | 224 × 224 | 28 × 28 | 64 × 64 | 128 × 128 | 224 × 224 |
| End-to-End | | | | | | | | |
| VGG16 | **94.79±0.02** | **94.82±0.01** | 94.79±0.06 | 94.83±0.01 | **76.16±0.31** | 78.59±0.11 | 80.07±0.26 | 80.92±0.11 |
| AlexNet | 94.75±0.01 | 94.77±0.00 | **94.80±0.02** | 94.79±0.01 | 72.31±0.20 | 74.75±0.29 | 77.21±0.32 | 78.87±0.22 |
| ResNet-18 | 94.75±0.01 | 94.77±0.00 | 94.79±0.02 | 94.79±0.01 | 73.82±0.12 | 75.59±0.24 | 78.24±0.32 | 78.96±0.30 |
| DenseNet-121 | 94.77±0.02 | 94.79±0.02 | **94.80±0.03** | **94.84±0.03** | 75.93±0.09 | **78.70±0.38** | **81.21±0.10** | **82.22±0.14** |
| EfficientNet-B4 | 94.37±0.32 | 94.31±0.27 | 94.76±0.02 | 94.76±0.03 | 67.44±1.87 | 71.63±0.64 | 77.65±0.35 | 78.25±0.31 |
| ViT-B/16 | 94.74±0.00 | 94.74±0.03 | 94.78±0.02 | 94.79±0.05 | 73.74±0.05 | 76.21±0.09 | 78.05±0.18 | 80.08±0.14 |
| CLIP ViT-B/16 | 94.74±0.01 | 94.75±0.00 | 94.76±0.01 | 94.74±0.06 | 72.44±0.35 | 73.20±0.42 | 74.18±0.23 | 77.77±0.42 |
| EVA-02 ViT-B/16 | 94.73±0.01 | 94.75±0.01 | 94.76±0.01 | 94.76±0.01 | 72.44±0.33 | 74.13±0.28 | 75.23±0.68 | 76.52±0.40 |
| DINO ViT-B/16 | 94.74±0.01 | 94.73±0.03 | 94.76±0.01 | 94.78±0.02 | 73.23±0.21 | 75.13±0.27 | 76.81±0.26 | 78.99±0.22 |
| SAM ViT-B/16 | 94.73±0.01 | 94.74±0.01 | 94.73±0.01 | 94.77±0.00 | 72.68±0.19 | 72.27±0.72 | 74.13±0.31 | 75.44±0.23 |
| Linear Probing | | | | | | | | |
| VGG16 | **94.75±0.00** | 94.75±0.00 | 94.75±0.00 | **94.76±0.00** | 69.04±0.02 | 70.45±0.03 | 73.69±0.04 | 73.91±0.06 |
| AlexNet | 94.74±0.00 | 94.74±0.00 | 94.75±0.00 | **94.76±0.00** | 61.61±0.36 | 66.60±0.31 | 71.49±0.18 | 74.37±0.04 |
| ResNet-18 | 94.74±0.00 | 94.74±0.00 | 94.74±0.00 | 94.74±0.00 | 61.51±0.07 | 62.56±0.36 | 66.63±0.18 | 75.12±0.01 |
| DenseNet-121 | 94.74±0.00 | 94.74±0.00 | 94.73±0.00 | 94.75±0.00 | 63.14±0.07 | 68.16±0.13 | 71.66±0.04 | 76.26±0.02 |
| EfficientNet-B4 | 94.68±0.02 | 94.69±0.03 | 94.72±0.01 | 94.74±0.00 | 55.93±2.16 | 58.77±4.36 | 68.20±0.30 | 74.89±0.01 |
| ViT-B/16 | 94.74±0.00 | 94.73±0.00 | 94.74±0.00 | 94.73±0.00 | 68.04±0.03 | 71.37±0.11 | 74.67±0.18 | 76.09±0.07 |
| CLIP ViT-B/16 | **94.75±0.00** | 94.72±0.00 | 94.74±0.00 | 94.75±0.00 | 70.65±0.03 | 71.22±0.01 | 74.41±0.15 | 76.29±0.15 |
| EVA-02 ViT-B/16 | 94.74±0.00 | 94.74±0.00 | 94.73±0.00 | 94.74±0.00 | 68.87±0.01 | 70.89±0.01 | 70.65±0.22 | 72.11±0.22 |
| DINO ViT-B/16 | **94.75±0.00** | **94.76±0.00** | **94.76±0.00** | **94.76±0.01** | **71.69±0.20** | **74.41±0.03** | **77.90±0.06** | **78.88±0.05** |
| SAM ViT-B/16 | 94.74±0.00 | 94.74±0.00 | 94.74±0.00 | 94.74±0.00 | 60.42±0.03 | 60.24±0.01 | 61.11±0.04 | 63.71±0.02 |
| $k$-NN ($k = 11$) | | | | | | | | |
| VGG16 | **94.67** | 94.66 | 94.66 | 94.67 | - | - | - | - |
| AlexNet | 94.66 | 94.68 | 94.67 | 94.66 | - | - | - | - |
| ResNet-18 | 94.66 | 94.68 | 94.66 | 94.66 | - | - | - | - |
| DenseNet-121 | 94.66 | 94.67 | 94.66 | 94.66 | - | - | - | - |
| EfficientNet-B4 | 94.65 | 94.67 | 94.68 | 94.67 | - | - | - | - |
| ViT-B/16 | 94.66 | 94.66 | **94.69** | **94.68** | - | - | - | - |
| CLIP ViT-B/16 | 94.66 | 94.66 | 94.67 | 94.67 | - | - | - | - |
| EVA-02 ViT-B/16 | 94.64 | 94.67 | 94.68 | 94.66 | - | - | - | - |
| DINO ViT-B/16 | 94.63 | 94.66 | 94.67 | 94.64 | - | - | - | - |
| SAM ViT-B/16 | 94.64 | **94.69** | **94.69** | **94.68** | - | - | - | - |

**Supplementary Table S4.** Benchmark outcomes summarizing the mean and standard deviation of accuracy (ACC), for a fixed operating point of 0.5, and area under the receiver operating characteristic curve (AUC) for the DermaMNIST dataset across all training scheme-model-image resolution combinations, derived from three independent random seeds. Notably, the $k$-NN algorithm, devoid of a training phase, remains unaffected by the stochasticity inherent in model training, thus reporting only the total ACC value without standard deviation. Moreover, owing to its direct utilization of embeddings and labels for classification, $k$-NN does not furnish a reliable AUC score. The overall best result across all training schemes, models, and resolutions is highlighted with a background color ; the best result per resolution across all training schemes and models is highlighted with underline; and the best result per training scheme and resolution is highlighted in **bold**.

| **DermaMNIST** | | | | | | | | |
|---|---|---|---|---|---|---|---|---|
| Methods | Accuracy (ACC) | | | | Area Under the ROC Curve (AUC) | | | |
| | $28 \times 28$ | $64 \times 64$ | $128 \times 128$ | $224 \times 224$ | $28 \times 28$ | $64 \times 64$ | $128 \times 128$ | $224 \times 224$ |
| END-TO-END | | | | | | | | |
| VGG16 | **76.58±0.26** | **79.53±1.19** | **81.80±1.29** | 81.55±1.89 | **92.69±0.59** | **94.36±0.40** | 95.71±0.46 | 95.51±0.69 |
| AlexNet | 76.13±0.35 | 78.69±0.18 | 80.42±0.75 | 82.04±1.19 | 92.23±0.21 | 94.34±0.06 | 95.13±0.25 | 96.10±0.09 |
| ResNet-18 | 73.38±0.61 | 76.36±0.22 | 79.19±0.51 | 82.33±0.36 | 88.35±0.28 | 91.70±0.76 | 93.46±0.39 | 95.27±0.27 |
| DenseNet-121 | 74.16±0.65 | 76.16±0.58 | 81.76±0.25 | **84.74±0.51** | 91.11±0.61 | 93.05±0.24 | 95.71±0.19 | 96.26±0.06 |
| EfficientNet-B4 | 68.74±0.75 | 71.45±0.10 | 73.83±0.30 | 76.38±1.29 | 83.87±0.66 | 87.58±0.27 | 89.32±0.16 | 91.88±0.85 |
| ViT-B/16 | 74.40±1.51 | 77.01±1.45 | 80.81±0.89 | 82.31±1.36 | 90.62±2.21 | 93.77±0.80 | **95.89±0.30** | 96.28±0.67 |
| CLIP ViT-B/16 | 72.97±0.15 | 72.44±0.19 | 74.73±0.42 | 75.31±0.28 | 90.32±0.12 | 91.55±0.45 | 92.51±0.14 | 92.59±0.26 |
| EVA-02 ViT-B/16 | 73.48±0.65 | 75.28±0.98 | 76.41±0.46 | 77.94±0.29 | 90.17±0.34 | 91.47±0.34 | 92.63±0.33 | 93.23±0.33 |
| DINO ViT-B/16 | 74.40±0.80 | 76.87±0.12 | 79.22±1.56 | 81.31±1.05 | 91.60±0.32 | 93.57±0.17 | 95.34±0.41 | **96.50±0.51** |
| SAM ViT-B/16 | 73.08±0.65 | 74.68±0.10 | 76.71±1.06 | 77.42±0.17 | 87.97±0.65 | 88.33±0.58 | 90.66±1.36 | 92.60±0.65 |
| LINEAR PROBING | | | | | | | | |
| VGG16 | 72.15±0.05 | 73.77±0.11 | 75.38±0.16 | 75.99±0.13 | 87.61±0.01 | 88.71±0.01 | 90.53±0.01 | 92.03±0.01 |
| AlexNet | 72.40±0.12 | 75.41±0.11 | 77.11±0.15 | 78.90±0.23 | 88.77±0.02 | 91.33±0.04 | 92.81±0.03 | 94.01±0.06 |
| ResNet-18 | 67.83±0.00 | 68.94±0.10 | 70.57±0.14 | 71.19±0.12 | 84.09±0.05 | 85.41±0.08 | 86.44±0.01 | 88.38±0.02 |
| DenseNet-121 | 71.70±0.06 | 74.26±0.15 | 77.39±0.12 | 77.06±0.18 | 89.80±0.03 | 90.94±0.08 | 92.15±0.05 | 93.18±0.04 |
| EfficientNet-B4 | 69.99±0.02 | 72.77±0.04 | 72.97±0.04 | 73.23±0.06 | 84.83±0.04 | 88.53±0.04 | 89.70±0.01 | 90.51±0.01 |
| ViT-B/16 | 72.15±0.15 | 77.64±0.24 | 80.90±0.25 | 82.01±0.02 | 89.89±0.04 | 93.58±0.04 | 95.05±0.02 | 95.88±0.03 |
| CLIP ViT-B/16 | 74.15±0.34 | 77.22±0.08 | 80.28±0.31 | 81.93±0.31 | 90.08±0.05 | 93.40±0.05 | 94.89±0.03 | 95.92±0.06 |
| EVA-02 ViT-B/16 | 73.47±0.12 | 75.54±0.12 | 77.17±0.10 | 79.29±0.02 | 90.50±0.05 | 92.76±0.04 | 93.88±0.05 | 94.69±0.03 |
| DINO ViT-B/16 | **75.78±0.17** | **79.88±0.68** | **81.65±0.66** | **84.42±0.23** | **91.87±0.10** | **95.40±0.20** | **95.95±0.17** | **96.83±0.07** |
| SAM ViT-B/16 | 66.88±0.00 | 66.88±0.00 | 66.88±0.00 | 66.88±0.00 | 66.73±0.88 | 70.87±0.37 | 72.64±0.46 | 69.34±0.33 |
| $k$-NN ($k = 11$) | | | | | | | | |
| VGG16 | 70.27 | 72.27 | 72.57 | 71.67 | - | - | - | - |
| AlexNet | 70.62 | 73.92 | 74.16 | 74.66 | - | - | - | - |
| ResNet-18 | 70.72 | 71.52 | 71.37 | 73.07 | - | - | - | - |
| DenseNet-121 | 69.33 | 70.62 | 72.67 | 73.17 | - | - | - | - |
| EfficientNet-B4 | 69.48 | 71.62 | 71.87 | 71.72 | - | - | - | - |
| ViT-B/16 | 69.43 | 70.87 | 72.62 | 75.21 | - | - | - | - |
| CLIP ViT-B/16 | 72.12 | 71.17 | 73.52 | 74.46 | - | - | - | - |
| EVA-02 ViT-B/16 | 73.22 | 73.77 | 74.11 | 75.61 | - | - | - | - |
| DINO ViT-B/16 | **73.97** | **75.91** | **76.51** | **78.35** | - | - | - | - |
| SAM ViT-B/16 | 69.73 | 70.42 | 70.22 | 68.38 | - | - | - | - |

**Supplementary Table S5.** Benchmark outcomes summarizing the mean and standard deviation of accuracy (ACC), for a fixed operating point of 0.5, and area under the receiver operating characteristic curve (AUC) for the OctMNIST dataset across all training scheme-model-image resolution combinations, derived from three independent random seeds. Notably, the *k*-NN algorithm, devoid of a training phase, remains unaffected by the stochasticity inherent in model training, thus reporting only the total ACC value without standard deviation. Moreover, owing to its direct utilization of embeddings and labels for classification, *k*-NN does not furnish a reliable AUC score. The overall best result across all training schemes, models, and resolutions is highlighted with a background color; the best result per resolution across all training schemes and models is highlighted with underline; and the best result per training scheme and resolution is highlighted in **bold**.

| OctMNIST | | | | | | | | |
|---|---|---|---|---|---|---|---|---|
| Methods | Accuracy (ACC) | | | | Area Under the ROC Curve (AUC) | | | |
| | $28 \times 28$ | $64 \times 64$ | $128 \times 128$ | $224 \times 224$ | $28 \times 28$ | $64 \times 64$ | $128 \times 128$ | $224 \times 224$ |
| END-TO-END | | | | | | | | |
| VGG16 | **77.50±2.86** | 81.93±2.44 | **90.50±0.85** | **90.30±2.62** | **95.84±1.00** | 97.50±0.79 | 98.73±0.26 | 99.17±0.13 |
| AlexNet | 66.40±1.13 | 75.47±2.28 | 80.57±1.90 | 84.63±1.73 | 90.96±0.39 | 95.09±0.76 | 97.92±0.22 | 98.01±0.48 |
| ResNet-18 | 69.07±0.83 | 80.70±2.01 | 84.03±1.97 | 85.10±1.12 | 92.10±0.36 | 97.53±0.26 | 97.77±0.58 | 98.81±0.28 |
| DenseNet-121 | 72.87±2.17 | **84.40±1.93** | 89.83±3.65 | 86.63±0.42 | 94.35±0.83 | **98.00±0.40** | 98.60±0.61 | **99.26±0.24** |
| EfficientNet-B4 | 59.93±2.79 | 75.87±1.55 | 80.73±2.00 | 82.57±4.67 | 88.65±0.74 | 95.61±0.08 | 98.09±0.12 | 98.93±0.24 |
| ViT-B/16 | 64.00±0.33 | 80.23±0.58 | 87.47±2.21 | 90.13±0.39 | 88.59±0.47 | 96.51±0.22 | **98.95±0.22** | 99.12±0.15 |
| CLIP ViT-B/16 | 61.53±0.70 | 77.73±1.78 | 83.43±3.42 | 86.80±3.07 | 87.46±0.13 | 95.68±0.03 | 98.11±0.58 | 98.88±0.41 |
| EVA-02 ViT-B/16 | 60.63±0.98 | 73.00±2.79 | 84.33±1.32 | 87.43±1.11 | 86.60±0.94 | 94.24±0.79 | 98.13±0.63 | 98.93±0.18 |
| DINO ViT-B/16 | 64.53±1.03 | 78.40±1.49 | 84.03±1.27 | 85.07±2.23 | 89.26±0.70 | 96.39±0.17 | 98.22±0.51 | 98.57±0.29 |
| SAM ViT-B/16 | 64.87±2.38 | 80.07±0.58 | 87.50±1.19 | 87.30±0.88 | 89.26±0.24 | 96.83±0.52 | 98.87±0.32 | 99.19±0.13 |
| LINEAR PROBING | | | | | | | | |
| VGG16 | 50.03±0.05 | 58.90±0.08 | 70.77±0.25 | 67.30±0.36 | 84.59±0.01 | 89.79±0.02 | 94.66±0.01 | 95.81±0.02 |
| AlexNet | 47.07±0.05 | 56.57±0.19 | 62.50±0.24 | 68.30±0.29 | 82.24±0.02 | 84.84±0.06 | 90.71±0.05 | 94.31±0.09 |
| ResNet-18 | 46.73±0.05 | 53.40±0.08 | 68.60±0.00 | 72.00±0.20 | 83.04±0.01 | 88.34±0.02 | 96.39±0.01 | 97.65±0.01 |
| DenseNet-121 | 56.17±0.12 | 66.07±0.24 | 71.10±0.33 | 78.47±0.34 | 88.87±0.01 | 94.37±0.01 | 97.27±0.01 | 98.72±0.01 |
| EfficientNet-B4 | 54.17±0.05 | 66.23±0.05 | 72.17±0.05 | 76.53±0.05 | 88.94±0.01 | 93.56±0.00 | 96.44±0.01 | 97.73±0.00 |
| ViT-B/16 | 54.43±0.05 | 66.80±0.33 | 76.87±0.12 | **83.57±0.12** | 86.31±0.01 | 94.94±0.01 | 97.22±0.04 | **98.96±0.03** |
| CLIP ViT-B/16 | 58.20±0.16 | 63.37±0.26 | **77.23±0.17** | 81.13±0.17 | 89.47±0.01 | 93.00±0.05 | 97.66±0.02 | 98.66±0.00 |
| EVA-02 ViT-B/16 | 53.33±0.17 | 60.27±0.05 | 63.87±0.19 | 68.50±0.24 | 87.44±0.02 | 92.72±0.02 | 95.95±0.01 | 96.47±0.01 |
| DINO ViT-B/16 | **62.63±0.09** | **71.20±0.49** | 75.47±0.21 | 73.73±0.99 | **92.30±0.04** | **95.99±0.11** | **97.83±0.04** | 98.35±0.08 |
| SAM ViT-B/16 | 26.20±0.00 | 28.87±0.05 | 34.50±0.00 | 40.80±0.00 | 66.00±0.05 | 72.17±0.06 | 71.19±0.03 | 80.36±0.03 |
| *k*-NN ($k = 11$) | | | | | | | | |
| VGG16 | 46.90 | 46.50 | 52.30 | 61.30 | - | - | - | - |
| AlexNet | 42.60 | 49.90 | 50.60 | 54.60 | - | - | - | - |
| ResNet-18 | 46.80 | 46.40 | 56.00 | 68.90 | - | - | - | - |
| DenseNet-121 | **49.20** | 50.30 | 55.30 | 63.20 | - | - | - | - |
| EfficientNet-B4 | 48.20 | 54.20 | 63.60 | 65.80 | - | - | - | - |
| ViT-B/16 | 46.70 | 48.70 | 58.90 | 65.70 | - | - | - | - |
| CLIP ViT-B/16 | 47.30 | 52.30 | 61.40 | 58.90 | - | - | - | - |
| EVA-02 ViT-B/16 | 46.00 | 51.20 | 55.50 | 58.90 | - | - | - | - |
| DINO ViT-B/16 | 46.50 | **61.10** | **72.20** | **74.10** | - | - | - | - |
| SAM ViT-B/16 | 39.30 | 39.10 | 40.50 | 44.00 | - | - | - | - |

**Supplementary Table S6.** Benchmark outcomes summarizing the mean and standard deviation of accuracy (ACC), for a fixed operating point of 0.5, and area under the receiver operating characteristic curve (AUC) for the OrganAMNIST dataset across all training scheme-model-image resolution combinations, derived from three independent random seeds. Notably, the $k$-NN algorithm, devoid of a training phase, remains unaffected by the stochasticity inherent in model training, thus reporting only the total ACC value without standard deviation. Moreover, owing to its direct utilization of embeddings and labels for classification, $k$-NN does not furnish a reliable AUC score. The overall best result across all training schemes, models, and resolutions is highlighted with a background color; the best result per resolution across all training schemes and models is highlighted with underline; and the best result per training scheme and resolution is highlighted in **bold**.

| **OrganAMNIST** | | | | | | | | |
|---|---|---|---|---|---|---|---|---|
| Methods | Accuracy (ACC) | | | | Area Under the ROC Curve (AUC) | | | |
| | $28 \times 28$ | $64 \times 64$ | $128 \times 128$ | $224 \times 224$ | $28 \times 28$ | $64 \times 64$ | $128 \times 128$ | $224 \times 224$ |
| END-TO-END | | | | | | | | |
| VGG16 | **93.19±0.49** | **96.44±0.35** | 94.24±1.94 | 94.61±0.64 | 99.47±0.05 | 99.79±0.04 | 99.75±0.12 | 99.75±0.05 |
| AlexNet | 90.87±0.21 | 95.23±0.38 | 95.34±0.33 | 95.93±0.29 | 99.37±0.03 | 99.78±0.06 | 99.79±0.03 | 99.85±0.04 |
| ResNet-18 | 92.04±0.04 | 95.49±0.12 | 96.21±0.30 | 96.01±0.11 | 99.30±0.12 | 99.76±0.05 | 99.84±0.02 | 99.70±0.02 |
| DenseNet-121 | 91.62±0.64 | 96.16±0.29 | **96.51±0.14** | 96.72±0.29 | **99.52±0.02** | **99.84±0.02** | **99.88±0.01** | 99.84±0.05 |
| EfficientNet-B4 | 85.81±1.30 | 93.52±0.67 | 95.58±0.23 | 95.11±0.27 | 98.76±0.09 | 99.65±0.08 | 99.85±0.02 | 99.79±0.01 |
| ViT-B/16 | 90.29±0.62 | 94.78±0.94 | 96.27±0.55 | **96.93±0.43** | 99.20±0.07 | 99.80±0.08 | 99.86±0.03 | **99.91±0.02** |
| CLIP ViT-B/16 | 88.10±0.23 | 93.90±0.34 | 94.83±0.43 | 95.25±0.39 | 98.97±0.06 | 99.70±0.06 | 99.81±0.03 | 99.81±0.05 |
| EVA-02 ViT-B/16 | 88.54±0.47 | 93.66±1.30 | 95.93±0.56 | 96.12±0.22 | 98.94±0.14 | 99.57±0.15 | 99.84±0.03 | 99.88±0.01 |
| DINO ViT-B/16 | 89.98±0.05 | 94.99±0.37 | 95.96±0.57 | 96.13±0.32 | 99.31±0.05 | 99.81±0.04 | 99.87±0.03 | 99.90±0.02 |
| SAM ViT-B/16 | 90.41±0.32 | 94.42±0.39 | 95.96±0.29 | 95.60±0.19 | 99.00±0.08 | 99.59±0.07 | 99.75±0.03 | 99.78±0.02 |
| LINEAR PROBING | | | | | | | | |
| VGG16 | 79.36±0.09 | 85.62±0.37 | 89.09±0.08 | 91.46±0.04 | 97.49±0.02 | 98.75±0.06 | 99.25±0.01 | 99.53±0.00 |
| AlexNet | 79.87±0.50 | 90.03±0.09 | 92.32±0.16 | 93.32±0.08 | 97.52±0.08 | 99.36±0.00 | 99.58±0.02 | 99.67±0.00 |
| ResNet-18 | 70.37±0.04 | 84.38±0.06 | 89.30±0.06 | 90.20±0.05 | 94.85±0.00 | 98.43±0.00 | 99.20±0.00 | 99.35±0.00 |
| DenseNet-121 | 81.99±0.08 | 90.71±0.04 | 92.73±0.05 | 93.63±0.10 | 98.03±0.02 | 99.42±0.01 | 99.67±0.00 | 99.73±0.01 |
| EfficientNet-B4 | 74.23±0.02 | 86.98±0.03 | 90.13±0.15 | 90.79±0.04 | 96.15±0.00 | 98.95±0.01 | 99.35±0.01 | 99.38±0.01 |
| ViT-B/16 | 81.45±0.43 | 90.21±0.15 | 92.14±0.06 | 93.06±0.36 | 97.49±0.08 | 99.42±0.02 | 99.59±0.00 | 99.65±0.03 |
| CLIP ViT-B/16 | 80.36±0.06 | 88.20±0.11 | 90.19±0.12 | 90.96±0.08 | 97.62±0.01 | 99.19±0.02 | 99.42±0.01 | 99.45±0.01 |
| EVA-02 ViT-B/16 | 81.68±0.29 | 87.12±0.05 | 88.50±0.10 | 89.97±0.20 | 97.92±0.03 | 98.93±0.01 | 99.18±0.01 | 99.37±0.03 |
| DINO ViT-B/16 | **89.74±0.37** | **93.91±0.16** | **94.97±0.13** | **94.96±0.02** | **99.27±0.04** | **99.74±0.01** | **99.79±0.01** | **99.78±0.01** |
| SAM ViT-B/16 | 22.54±0.07 | 39.10±0.25 | 61.65±0.08 | 71.18±0.05 | 79.07±0.05 | 90.11±0.02 | 94.50±0.01 | 95.29±0.01 |
| $k$-NN ($k = 11$) | | | | | | | | |
| VGG16 | 70.42 | 80.40 | 82.90 | 84.55 | - | - | - | - |
| AlexNet | 72.71 | 82.87 | 86.54 | 88.38 | - | - | - | - |
| ResNet-18 | 69.68 | 81.25 | 86.08 | 86.69 | - | - | - | - |
| DenseNet-121 | 69.48 | 81.93 | 86.73 | 87.28 | - | - | - | - |
| EfficientNet-B4 | 69.92 | 81.16 | 83.19 | 82.15 | - | - | - | - |
| ViT-B/16 | 66.71 | 80.67 | 83.13 | 83.86 | - | - | - | - |
| CLIP ViT-B/16 | 66.65 | 79.68 | 81.29 | 82.77 | - | - | - | - |
| EVA-02 ViT-B/16 | 73.33 | 80.74 | 82.71 | 83.59 | - | - | - | - |
| DINO ViT-B/16 | **84.59** | **90.75** | **91.25** | **90.69** | - | - | - | - |
| SAM ViT-B/16 | 70.08 | 82.34 | 83.67 | 83.14 | - | - | - | - |

**Supplementary Table S7.** Benchmark outcomes summarizing the mean and standard deviation of accuracy (ACC), for a fixed operating point of 0.5, and area under the receiver operating characteristic curve (AUC) for the OrganCMNIST dataset across all training scheme-model-image resolution combinations, derived from three independent random seeds. Notably, the $k$-NN algorithm, devoid of a training phase, remains unaffected by the stochasticity inherent in model training, thus reporting only the total ACC value without standard deviation. Moreover, owing to its direct utilization of embeddings and labels for classification, $k$-NN does not furnish a reliable AUC score. The overall best result across all training schemes, models, and resolutions is highlighted with a background color ; the best result per resolution across all training schemes and models is highlighted with underline; and the best result per training scheme and resolution is highlighted in **bold**.

| **OrganCMNIST** | | | | | | | | |
|---|---|---|---|---|---|---|---|---|
| Methods | Accuracy (ACC) | | | | Area Under the ROC Curve (AUC) | | | |
| | $28 \times 28$ | $64 \times 64$ | $128 \times 128$ | $224 \times 224$ | $28 \times 28$ | $64 \times 64$ | $128 \times 128$ | $224 \times 224$ |
| END-TO-END | | | | | | | | |
| VGG16 | 91.35±0.41 | 93.51±0.72 | 92.54±0.88 | 93.37±0.34 | **99.32±0.08** | 99.64±0.05 | 99.61±0.03 | 99.65±0.02 |
| AlexNet | 87.76±1.73 | 92.82±0.25 | 93.84±0.20 | 93.17±0.16 | 99.13±0.16 | 99.61±0.02 | 99.72±0.03 | 99.71±0.03 |
| ResNet-18 | 90.48±0.42 | 93.25±0.27 | 93.94±0.31 | 93.20±0.12 | 99.14±0.03 | 99.60±0.06 | 99.68±0.02 | 99.61±0.07 |
| DenseNet-121 | **91.52±0.33** | **93.99±0.32** | **94.42±0.37** | 93.72±0.52 | 99.27±0.05 | **99.69±0.02** | 99.65±0.05 | 99.67±0.02 |
| EfficientNet-B4 | 84.25±1.79 | 89.15±2.09 | 90.93±0.70 | 90.60±0.16 | 98.39±0.17 | 99.17±0.23 | 99.50±0.05 | 99.46±0.01 |
| ViT-B/16 | 89.34±1.00 | 93.20±0.53 | 93.34±0.46 | **94.02±0.45** | 99.13±0.08 | 99.71±0.02 | 99.72±0.05 | **99.78±0.04** |
| CLIP ViT-B/16 | 87.52±0.25 | 90.88±0.84 | 91.54±1.63 | 92.39±0.43 | 98.94±0.03 | 99.40±0.02 | 99.53±0.15 | 99.61±0.08 |
| EVA-02 ViT-B/16 | 88.77±0.56 | 91.86±0.59 | 93.93±0.34 | **94.02±0.84** | 98.77±0.16 | 99.33±0.08 | 99.61±0.09 | 99.61±0.05 |
| DINO ViT-B/16 | 89.51±0.75 | 92.36±0.09 | 93.78±1.39 | 93.68±0.14 | 99.24±0.01 | 99.63±0.02 | **99.75±0.09** | **99.78±0.02** |
| SAM ViT-B/16 | 89.04±0.54 | 92.94±0.05 | 92.12±1.13 | 92.71±0.51 | 98.63±0.17 | 99.31±0.09 | 99.33±0.13 | 99.39±0.10 |
| LINEAR PROBING | | | | | | | | |
| VGG16 | 75.62±0.28 | 81.49±0.21 | 84.30±0.36 | 85.11±0.11 | 96.67±0.05 | 98.03±0.03 | 98.58±0.05 | 98.81±0.01 |
| AlexNet | 78.15±0.50 | 87.39±0.13 | 88.66±0.12 | 89.74±0.14 | 97.32±0.10 | 98.97±0.02 | 99.16±0.01 | 99.20±0.01 |
| ResNet-18 | 60.70±0.02 | 75.86±0.12 | 81.65±0.03 | 82.21±0.01 | 92.56±0.01 | 97.04±0.00 | 98.20±0.00 | 98.33±0.00 |
| DenseNet-121 | 76.22±0.10 | 85.80±0.09 | 87.70±0.10 | 88.09±0.13 | 97.01±0.00 | 98.88±0.00 | 99.14±0.01 | 99.21±0.01 |
| EfficientNet-B4 | 65.08±0.03 | 78.61±0.06 | 81.67±0.07 | 83.85±0.07 | 93.77±0.00 | 97.70±0.00 | 98.27±0.00 | 98.59±0.00 |
| ViT-B/16 | 77.36±0.30 | 85.46±0.14 | 90.82±5.27 | 88.20±0.05 | 97.22±0.04 | 98.92±0.01 | 99.06±0.02 | 99.19±0.00 |
| CLIP ViT-B/16 | 76.78±0.11 | 83.04±0.20 | 83.93±0.13 | 84.12±0.19 | 96.93±0.02 | 98.54±0.03 | 98.68±0.00 | 98.72±0.03 |
| EVA-02 ViT-B/16 | 78.51±0.04 | 81.63±0.29 | 83.60±0.34 | 83.85±0.10 | 97.41±0.00 | 98.36±0.05 | 98.66±0.04 | 98.66±0.00 |
| DINO ViT-B/16 | **88.34±0.06** | **92.21±0.12** | **91.82±0.22** | **92.46±1.70** | **99.08±0.01** | **99.62±0.01** | **99.60±0.02** | **99.54±0.01** |
| SAM ViT-B/16 | 22.33±0.00 | 22.33±0.00 | 32.01±0.11 | 58.60±0.10 | 62.32±3.37 | 87.56±0.08 | 93.09±0.02 | 93.31±0.00 |
| $k$-NN ($k = 11$) | | | | | | | | |
| VGG16 | 67.70 | 75.21 | 77.31 | 75.65 | - | - | - | - |
| AlexNet | 72.47 | 80.22 | 81.86 | 83.00 | - | - | - | - |
| ResNet-18 | 66.20 | 76.56 | 80.00 | 80.08 | - | - | - | - |
| DenseNet-121 | 62.97 | 73.03 | 78.44 | 79.99 | - | - | - | - |
| EfficientNet-B4 | 63.83 | 74.25 | 72.69 | 73.47 | - | - | - | - |
| ViT-B/16 | 64.74 | 72.59 | 76.06 | 78.25 | - | - | - | - |
| CLIP ViT-B/16 | 59.30 | 70.87 | 73.69 | 74.66 | - | - | - | - |
| EVA-02 ViT-B/16 | 72.48 | 75.23 | 76.64 | 77.57 | - | - | - | - |
| DINO ViT-B/16 | **83.63** | **87.52** | **87.13** | **86.00** | - | - | - | - |
| SAM ViT-B/16 | 71.15 | 82.05 | 84.07 | 83.80 | - | - | - | - |

**Supplementary Table S8.** Benchmark outcomes summarizing the mean and standard deviation of accuracy (ACC), for a fixed operating point of 0.5, and area under the receiver operating characteristic curve (AUC) for the OrganSMNIST dataset across all training scheme-model-image resolution combinations, derived from three independent random seeds. Notably, the *k*-NN algorithm, devoid of a training phase, remains unaffected by the stochasticity inherent in model training, thus reporting only the total ACC value without standard deviation. Moreover, owing to its direct utilization of embeddings and labels for classification, *k*-NN does not furnish a reliable AUC score. The overall best result across all training schemes, models, and resolutions is highlighted with a background color; the best result per resolution across all training schemes and models is highlighted with underline; and the best result per training scheme and resolution is highlighted in **bold**.

| **OrganSMNIST** | | | | | | | | |
|---|---|---|---|---|---|---|---|---|
| Methods | Accuracy (ACC) | | | | Area Under the ROC Curve (AUC) | | | |
| | 28 × 28 | 64 × 64 | 128 × 128 | 224 × 224 | 28 × 28 | 64 × 64 | 128 × 128 | 224 × 224 |
| End-to-End | | | | | | | | |
| VGG16 | **78.87±0.68** | 81.60±0.21 | 82.36±0.08 | 82.06±0.48 | **97.64±0.09** | 98.06±0.24 | 98.11±0.06 | 97.89±0.18 |
| AlexNet | 76.75±0.44 | 80.58±0.90 | 81.71±0.17 | 81.56±0.36 | 97.30±0.13 | 97.87±0.17 | 98.07±0.06 | 98.17±0.03 |
| ResNet-18 | 76.24±0.36 | 80.34±0.82 | 82.30±0.29 | 81.24±0.42 | 97.23±0.15 | 97.91±0.07 | 98.19±0.13 | 97.86±0.15 |
| DenseNet-121 | 77.70±0.61 | **83.47±0.24** | **83.18±0.19** | 81.80±0.66 | 97.17±0.19 | 97.93±0.05 | 97.94±0.13 | 98.11±0.16 |
| EfficientNet-B4 | 67.97±0.79 | 76.01±0.73 | 77.33±0.38 | 76.37±0.42 | 95.07±0.20 | 97.17±0.08 | 97.50±0.04 | 97.33±0.03 |
| ViT-B/16 | 76.45±0.83 | 81.43±1.28 | 82.94±0.33 | **82.50±0.60** | 97.12±0.03 | **98.19±0.19** | **98.50±0.08** | 98.31±0.18 |
| CLIP ViT-B/16 | 73.35±0.31 | 78.08±2.76 | 79.65±1.72 | 78.69±1.71 | 96.53±0.19 | 97.56±0.33 | 97.96±0.15 | 97.76±0.11 |
| EVA-02 ViT-B/16 | 71.91±3.07 | 78.77±1.99 | 81.19±1.35 | 81.62±0.80 | 95.80±0.65 | 97.49±0.24 | 98.02±0.15 | 98.09±0.11 |
| DINO ViT-B/16 | 76.10±0.85 | 79.38±2.39 | 82.72±0.33 | 81.72±0.57 | 97.09±0.18 | 98.00±0.12 | 98.33±0.05 | **98.33±0.05** |
| SAM ViT-B/16 | 76.71±0.41 | 80.18±0.19 | 80.61±0.54 | 81.00±0.63 | 96.11±0.14 | 97.34±0.15 | 97.48±0.21 | 97.70±0.10 |
| Linear Probing | | | | | | | | |
| VGG16 | 63.33±0.23 | 68.83±0.28 | 72.08±0.23 | 72.85±0.07 | 93.70±0.07 | 95.48±0.06 | 96.37±0.01 | 96.62±0.00 |
| AlexNet | 63.75±0.77 | 72.14±0.38 | 74.91±0.04 | 75.65±0.18 | 94.44±0.16 | 96.73±0.06 | 97.24±0.00 | 97.12±0.00 |
| ResNet-18 | 53.65±0.06 | 64.95±0.11 | 70.19±0.04 | 70.12±0.06 | 89.55±0.00 | 94.31±0.01 | 95.78±0.01 | 95.84±0.00 |
| DenseNet-121 | 66.28±0.03 | 74.96±0.06 | 76.10±0.12 | 76.56±0.19 | 94.86±0.00 | 96.93±0.02 | 97.32±0.02 | 97.41±0.02 |
| EfficientNet-B4 | 58.50±0.05 | 70.32±0.03 | 71.35±0.03 | 72.06±0.07 | 91.96±0.00 | 95.72±0.01 | 96.16±0.01 | 96.44±0.00 |
| ViT-B/16 | 65.44±0.15 | 74.63±0.28 | 77.41±0.24 | 77.45±0.22 | 94.46±0.01 | 97.05±0.02 | 97.48±0.05 | 97.55±0.01 |
| CLIP ViT-B/16 | 65.39±0.07 | 72.82±0.07 | 74.67±0.15 | 74.77±0.13 | 94.23±0.01 | 96.61±0.00 | 96.87±0.04 | 96.93±0.02 |
| EVA-02 ViT-B/16 | 66.42±0.13 | 69.52±0.05 | 73.08±0.09 | 74.24±0.09 | 95.01±0.02 | 96.21±0.02 | 96.54±0.02 | 96.91±0.01 |
| DINO ViT-B/16 | **74.25±0.36** | **78.70±0.06** | **80.79±0.24** | **79.27±0.17** | **97.03±0.02** | **97.92±0.00** | **98.09±0.00** | **97.89±0.01** |
| SAM ViT-B/16 | 23.54±0.00 | 23.54±0.00 | 35.64±0.02 | 49.05±0.03 | 55.66±5.45 | 56.44±3.28 | 89.56±0.02 | 89.66±0.02 |
| *k*-NN ($k = 11$) | | | | | | | | |
| VGG16 | 55.25 | 63.80 | 64.98 | 65.80 | - | - | - | - |
| AlexNet | 60.67 | 66.50 | 68.29 | 68.97 | - | - | - | - |
| ResNet-18 | 56.67 | 65.83 | 70.26 | 70.08 | - | - | - | - |
| DenseNet-121 | 55.61 | 64.96 | 67.96 | 69.56 | - | - | - | - |
| EfficientNet-B4 | 55.84 | 66.22 | 64.81 | 63.92 | - | - | - | - |
| ViT-B/16 | 52.02 | 66.04 | 69.30 | 69.81 | - | - | - | - |
| CLIP ViT-B/16 | 50.91 | 64.35 | 66.65 | 67.27 | - | - | - | - |
| EVA-02 ViT-B/16 | 60.61 | 65.29 | 65.62 | 68.81 | - | - | - | - |
| DINO ViT-B/16 | **70.69** | **76.64** | **77.70** | **74.82** | - | - | - | - |
| SAM ViT-B/16 | 56.72 | 67.13 | 70.62 | 69.77 | - | - | - | - |

**Supplementary Table S9.** Benchmark outcomes summarizing the mean and standard deviation of accuracy (ACC), for a fixed operating point of 0.5, and area under the receiver operating characteristic curve (AUC) for the PathMNIST dataset across all training scheme-model-image resolution combinations, derived from three independent random seeds. Notably, the *k*-NN algorithm, devoid of a training phase, remains unaffected by the stochasticity inherent in model training, thus reporting only the total ACC value without standard deviation. Moreover, owing to its direct utilization of embeddings and labels for classification, *k*-NN does not furnish a reliable AUC score. The overall best result across all training schemes, models, and resolutions is highlighted with a background color ; the best result per resolution across all training schemes and models is highlighted with underline; and the best result per training scheme and resolution is highlighted in **bold**.

| PathMNIST | | | | | | | | |
|---|---|---|---|---|---|---|---|---|
| Methods | Accuracy (ACC) | | | | Area Under the ROC Curve (AUC) | | | |
| | $28 \times 28$ | $64 \times 64$ | $128 \times 128$ | $224 \times 224$ | $28 \times 28$ | $64 \times 64$ | $128 \times 128$ | $224 \times 224$ |
| END-TO-END | | | | | | | | |
| VGG16 | **88.93±0.53** | **93.83±1.12** | 94.85±0.61 | 94.73±0.31 | **98.57±0.14** | 99.34±0.12 | 99.42±0.18 | 99.33±0.06 |
| AlexNet | 80.75±1.63 | 89.00±1.35 | 93.00±0.65 | 94.19±0.99 | 96.67±0.41 | 98.78±0.17 | 99.35±0.04 | 99.41±0.11 |
| ResNet-18 | 85.54±0.87 | 93.36±0.65 | 95.27±0.23 | 93.82±1.19 | 98.24±0.14 | 99.40±0.11 | 99.62±0.10 | 99.30±0.15 |
| DenseNet-121 | 85.26±0.36 | 92.34±0.84 | 94.69±0.31 | 95.74±0.58 | 98.18±0.14 | **99.43±0.06** | **99.70±0.06** | **99.79±0.06** |
| EfficientNet-B4 | 76.35±3.04 | 88.39±1.94 | 94.20±0.64 | 92.60±0.57 | 96.27±0.61 | 98.90±0.25 | 99.57±0.02 | 99.45±0.13 |
| ViT-B/16 | 82.82±0.29 | 91.97±0.39 | 94.61±0.90 | 95.82±0.25 | 97.81±0.31 | 99.28±0.11 | 99.67±0.08 | 99.64±0.10 |
| CLIP ViT-B/16 | 81.41±0.75 | 87.47±1.96 | 92.71±0.34 | 92.51±1.56 | 97.79±0.22 | 98.79±0.13 | 99.49±0.01 | 99.47±0.11 |
| EVA-02 ViT-B/16 | 82.33±1.71 | 90.54±1.68 | 94.88±1.02 | 95.97±0.85 | 97.69±0.16 | 99.17±0.19 | 99.69±0.10 | 99.75±0.10 |
| DINO ViT-B/16 | 82.23±1.32 | 90.29±1.80 | 94.24±0.23 | 94.33±0.90 | 97.56±0.38 | 99.13±0.07 | 99.68±0.03 | 99.54±0.22 |
| SAM ViT-B/16 | 84.45±0.53 | 91.82±1.60 | **95.67±1.27** | **96.07±0.41** | 98.10±0.23 | 99.37±0.10 | 99.66±0.02 | 99.76±0.05 |
| LINEAR PROBING | | | | | | | | |
| VGG16 | 80.04±0.01 | 84.88±0.05 | 87.00±0.04 | 87.84±0.06 | 96.79±0.00 | 98.21±0.00 | 98.50±0.00 | 98.76±0.01 |
| AlexNet | 76.95±0.04 | 81.48±0.06 | 86.86±0.14 | 88.21±0.14 | 95.33±0.00 | 96.87±0.02 | 98.59±0.01 | 98.79±0.03 |
| ResNet-18 | 73.53±0.01 | 84.42±0.02 | 88.12±0.02 | 88.94±0.02 | 95.42±0.00 | 98.05±0.00 | 98.57±0.00 | 98.88±0.00 |
| DenseNet-121 | 81.82±0.01 | 90.03±0.05 | 92.02±0.02 | 91.28±0.06 | 97.80±0.01 | 99.11±0.00 | 99.40±0.00 | 99.21±0.01 |
| EfficientNet-B4 | 80.51±0.03 | 86.45±0.02 | 87.62±0.02 | 89.67±0.02 | 97.33±0.00 | 98.74±0.00 | 98.84±0.00 | 98.88±0.00 |
| ViT-B/16 | 83.90±0.04 | 91.43±0.02 | 92.70±0.02 | 93.54±0.08 | 97.90±0.00 | 99.33±0.00 | 99.50±0.00 | 99.63±0.01 |
| CLIP ViT-B/16 | 83.78±0.02 | 90.83±0.08 | 91.12±0.17 | 91.82±0.07 | **98.01±0.00** | 99.17±0.00 | 99.33±0.00 | 99.32±0.01 |
| EVA-02 ViT-B/16 | 82.53±0.05 | 90.07±0.04 | 91.73±0.03 | 87.37±0.03 | 97.76±0.00 | 99.03±0.00 | 99.34±0.00 | 99.02±0.00 |
| DINO ViT-B/16 | **85.05±0.11** | **93.90±0.11** | **94.27±0.07** | **96.14±0.05** | 97.93±0.02 | **99.59±0.01** | **99.64±0.01** | **99.75±0.00** |
| SAM ViT-B/16 | 31.28±0.05 | 56.31±0.01 | 64.34±0.05 | 75.75±0.01 | 77.04±0.02 | 88.94±0.01 | 91.06±0.00 | 96.37±0.00 |
| *k*-NN ($k = 11$) | | | | | | | | |
| VGG16 | 70.32 | 76.82 | 78.84 | 82.19 | - | - | - | - |
| AlexNet | 71.52 | 74.55 | 81.23 | 84.21 | - | - | - | - |
| ResNet-18 | 68.89 | 79.25 | 81.85 | 83.47 | - | - | - | - |
| DenseNet-121 | 72.90 | 82.16 | 86.16 | 85.86 | - | - | - | - |
| EfficientNet-B4 | 73.84 | 80.45 | 81.25 | 80.45 | - | - | - | - |
| ViT-B/16 | 71.96 | 81.50 | 86.57 | 88.04 | - | - | - | - |
| CLIP ViT-B/16 | 73.48 | 83.02 | 85.58 | 86.49 | - | - | - | - |
| EVA-02 ViT-B/16 | 76.17 | 83.58 | 86.49 | 79.94 | - | - | - | - |
| DINO ViT-B/16 | **80.54** | **90.39** | **93.72** | **94.32** | - | - | - | - |
| SAM ViT-B/16 | 63.91 | 76.99 | 78.02 | 77.28 | - | - | - | - |

**Supplementary Table S10.** Benchmark outcomes summarizing the mean and standard deviation of accuracy (ACC), for a fixed operating point of 0.5, and area under the receiver operating characteristic curve (AUC) for the PneumoniaMNIST dataset across all training scheme-model-image resolution combinations, derived from three independent random seeds. Notably, the $k$-NN algorithm, devoid of a training phase, remains unaffected by the stochasticity inherent in model training, thus reporting only the total ACC value without standard deviation. Moreover, owing to its direct utilization of embeddings and labels for classification, $k$-NN does not furnish a reliable AUC score. The overall best result across all training schemes, models, and resolutions is highlighted with a background color ; the best result per resolution across all training schemes and models is highlighted with underline; and the best result per training scheme and resolution is highlighted in **bold**.

| **PneumoniaMNIST** | | | | | | | | |
|---|---|---|---|---|---|---|---|---|
| Methods | Accuracy (ACC) | | | | Area Under the ROC Curve (AUC) | | | |
| | $28 \times 28$ | $64 \times 64$ | $128 \times 128$ | $224 \times 224$ | $28 \times 28$ | $64 \times 64$ | $128 \times 128$ | $224 \times 224$ |
| END-TO-END | | | | | | | | |
| VGG16 | 84.78±2.55 | **89.26±0.39** | 87.13±1.93 | 87.39±1.00 | **97.04±0.54** | **98.69±0.16** | 98.55±0.44 | **98.47±0.16** |
| AlexNet | **85.10±0.73** | 88.03±1.64 | 87.18±1.67 | 87.23±0.77 | 95.88±0.42 | 96.71±0.64 | 97.85±0.32 | 98.25±0.13 |
| ResNet-18 | 83.12±1.10 | 86.00±1.44 | 89.85±1.37 | **91.13±0.96** | 95.01±0.30 | 94.34±0.71 | 97.91±0.23 | 98.04±0.30 |
| DenseNet-121 | 83.55±2.21 | 85.42±1.36 | 89.80±0.79 | 88.94±2.30 | 96.06±0.24 | 97.19±0.29 | **98.75±0.23** | 97.41±0.80 |
| EfficientNet-B4 | 79.01±1.44 | 82.69±0.13 | 85.15±0.93 | 87.13±1.14 | 90.69±0.46 | 95.34±0.52 | 96.91±0.21 | 96.71±0.72 |
| ViT-B/16 | 83.65±2.77 | 85.84±1.32 | 83.76±2.17 | 86.11±3.57 | 95.60±0.27 | 96.41±0.10 | 96.81±0.73 | 96.33±0.51 |
| CLIP ViT-B/16 | 84.56±1.96 | 84.62±1.04 | 84.62±1.73 | 83.81±1.83 | 94.66±0.36 | 94.63±0.19 | 94.61±0.75 | 95.59±1.30 |
| EVA-02 ViT-B/16 | 85.04±1.52 | 86.59±1.14 | 83.17±0.32 | 82.75±2.53 | 93.88±1.04 | 94.99±1.14 | 93.81±1.21 | 93.54±1.82 |
| DINO ViT-B/16 | 84.13±2.06 | 85.04±2.16 | **90.06±0.65** | 84.08±2.13 | 95.52±0.62 | 96.00±0.28 | 97.96±0.71 | 96.67±0.95 |
| SAM ViT-B/16 | 83.71±2.08 | 86.65±0.98 | 86.16±2.69 | 83.81±2.85 | 91.94±1.45 | 93.68±2.74 | 95.88±1.61 | 94.53±2.71 |
| LINEAR PROBING | | | | | | | | |
| VGG16 | 81.57±0.26 | 84.13±0.32 | 83.33±0.23 | 86.22±0.13 | 91.93±0.03 | 95.59±0.10 | 96.15±0.03 | 96.75±0.04 |
| AlexNet | 78.10±0.50 | 83.92±0.40 | 85.90±0.26 | 88.09±0.15 | 91.14±0.07 | 94.89±0.05 | 97.51±0.06 | 97.57±0.01 |
| ResNet-18 | 74.36±0.26 | 78.90±0.20 | 80.40±0.20 | 83.17±0.13 | 87.69±0.08 | 94.21±0.02 | 94.40±0.05 | 96.91±0.01 |
| DenseNet-121 | 81.94±0.20 | 82.85±0.13 | 84.88±0.27 | 86.59±0.20 | 92.68±0.14 | 96.01±0.06 | 96.53±0.05 | 97.34±0.05 |
| EfficientNet-B4 | 82.64±0.08 | 84.19±0.08 | 84.40±0.15 | 87.07±0.08 | 93.66±0.03 | 96.29±0.02 | 95.41±0.02 | 97.21±0.01 |
| ViT-B/16 | 83.12±0.20 | 84.51±0.72 | 87.50±0.35 | 88.30±0.26 | 94.28±0.02 | 95.93±0.11 | 97.33±0.03 | 97.63±0.07 |
| CLIP ViT-B/16 | 84.88±0.20 | 85.04±0.20 | 84.67±0.20 | 84.99±0.27 | 94.48±0.19 | 96.54±0.05 | 96.64±0.11 | 97.17±0.07 |
| EVA-02 ViT-B/16 | 83.60±0.08 | 79.22±0.15 | 81.52±0.08 | 83.65±0.13 | 94.10±0.03 | 94.30±0.01 | 95.42±0.03 | 96.34±0.05 |
| DINO ViT-B/16 | **86.59±0.54** | **86.43±0.65** | **90.33±0.15** | **91.56±0.27** | **97.29±0.07** | **97.56±0.10** | **98.89±0.03** | **98.92±0.08** |
| SAM ViT-B/16 | 62.50±0.00 | 62.50±0.00 | 62.50±0.00 | 62.50±0.00 | 81.25±0.50 | 87.16±0.25 | 92.52±0.05 | 90.13±0.14 |
| $k$-NN ($k = 11$) | | | | | | | | |
| VGG16 | 76.12 | 83.01 | 81.73 | 81.57 | - | - | - | - |
| AlexNet | 81.89 | 81.25 | 83.65 | 84.62 | - | - | - | - |
| ResNet-18 | 81.09 | 86.06 | 83.97 | 87.34 | - | - | - | - |
| DenseNet-121 | 82.05 | 82.37 | 84.29 | 86.06 | - | - | - | - |
| EfficientNet-B4 | 85.58 | 83.81 | 84.78 | 84.62 | - | - | - | - |
| ViT-B/16 | 83.01 | 77.88 | 84.94 | 87.82 | - | - | - | - |
| CLIP ViT-B/16 | 85.26 | 83.01 | 86.38 | 87.50 | - | - | - | - |
| EVA-02 ViT-B/16 | 84.29 | 79.81 | 83.97 | 84.94 | - | - | - | - |
| DINO ViT-B/16 | **85.74** | **87.82** | **90.54** | **89.74** | - | - | - | - |
| SAM ViT-B/16 | 80.93 | 83.33 | 84.13 | 86.06 | - | - | - | - |

**Supplementary Table S11.** Benchmark outcomes summarizing the mean and standard deviation of accuracy (ACC), for a fixed operating point of 0.5, and area under the receiver operating characteristic curve (AUC) for the RetinaMNIST dataset across all training scheme-model-image resolution combinations, derived from three independent random seeds. Notably, the $k$-NN algorithm, devoid of a training phase, remains unaffected by the stochasticity inherent in model training, thus reporting only the total ACC value without standard deviation. Moreover, owing to its direct utilization of embeddings and labels for classification, $k$-NN does not furnish a reliable AUC score. The overall best result across all training schemes, models, and resolutions is highlighted with a background color ; the best result per resolution across all training schemes and models is highlighted with underline; and the best result per training scheme and resolution is highlighted in **bold**.

| RetinaMNIST | | | | | | | | |
|---|---|---|---|---|---|---|---|---|
| Methods | Accuracy (ACC) | | | | Area Under the ROC Curve (AUC) | | | |
| | 28 × 28 | 64 × 64 | 128 × 128 | 224 × 224 | 28 × 28 | 64 × 64 | 128 × 128 | 224 × 224 |
| END-TO-END | | | | | | | | |
| VGG16 | **54.17±0.77** | **55.75±4.48** | **62.00±1.02** | **64.17±2.42** | **75.35±0.98** | **80.63±0.68** | **85.26±0.60** | **87.82±0.71** |
| AlexNet | 51.83±0.66 | 52.58±0.42 | 58.25±1.74 | 59.42±0.96 | 72.80±0.64 | 75.08±0.38 | 80.02±0.95 | 83.20±1.02 |
| ResNet-18 | 52.33±2.37 | 53.08±1.48 | 59.25±0.41 | 61.50±1.34 | 70.41±0.55 | 74.40±1.90 | 80.81±0.61 | 83.16±0.65 |
| DenseNet-121 | 48.67±0.51 | 53.25±2.15 | 61.75±0.20 | 61.75±1.08 | 71.17±1.16 | 74.30±0.74 | 81.45±0.51 | 82.90±0.57 |
| EfficientNet-B4 | 47.25±2.70 | 50.67±0.77 | 53.92±1.36 | 52.42±1.66 | 64.12±2.59 | 70.83±0.30 | 73.61±0.65 | 73.83±0.76 |
| ViT-B/16 | 49.83±1.53 | 54.08±0.96 | 54.00±2.27 | 55.08±2.05 | 71.72±0.89 | 73.58±1.65 | 73.71±0.81 | 78.58±2.32 |
| CLIP ViT-B/16 | 52.50±1.06 | 51.58±0.85 | 50.58±1.01 | 50.33±0.62 | 72.80±0.93 | 72.69±1.72 | 71.43±0.62 | 70.84±1.46 |
| EVA-02 ViT-B/16 | 51.25±1.22 | 51.67±0.24 | 47.67±3.37 | 54.42±1.53 | 71.23±0.91 | 71.15±1.19 | 69.91±2.11 | 74.73±2.80 |
| DINO ViT-B/16 | 52.33±1.64 | 50.83±1.90 | 50.33±2.01 | 54.25±2.41 | 73.32±0.28 | 71.91±0.70 | 71.96±0.60 | 78.32±3.37 |
| SAM ViT-B/16 | 50.00±1.95 | 50.67±2.71 | 51.17±1.05 | 51.33±1.45 | 71.25±1.60 | 71.64±1.66 | 71.90±0.96 | 71.72±1.39 |
| LINEAR PROBING | | | | | | | | |
| VGG16 | 50.58±0.24 | 53.42±1.01 | 57.33±0.24 | 61.08±0.42 | 71.84±0.31 | 75.50±0.17 | 81.06±0.18 | 84.86±0.27 |
| AlexNet | 51.25±0.35 | 54.25±0.74 | 56.17±0.47 | 58.08±0.12 | 70.94±0.17 | 74.07±0.32 | 78.31±0.1 | 81.33±0.20 |
| ResNet-18 | 43.50±0.00 | 46.58±0.24 | 47.50±0.20 | 49.50±0.20 | 68.91±0.52 | 71.18±0.35 | 75.52±0.16 | 79.45±0.21 |
| DenseNet-121 | 52.08±0.77 | 54.83±0.59 | **60.17±0.31** | **62.67±0.92** | **73.41±0.14** | 77.01±0.22 | **83.46±0.24** | **85.93±0.23** |
| EfficientNet-B4 | 51.83±0.42 | **57.42±0.42** | 58.58±0.12 | 58.75±0.20 | 72.51±0.14 | 76.23±0.07 | 80.23±0.07 | 81.32±0.07 |
| ViT-B/16 | **54.25±1.08** | 55.75±1.27 | 59.00±0.54 | 61.17±0.72 | 73.09±0.07 | 75.15±0.32 | 82.47±0.52 | 85.16±0.55 |
| CLIP ViT-B/16 | 54.17±1.45 | 56.00±1.41 | 59.33±0.66 | 61.25±0.54 | 73.10±0.29 | **77.77±0.55** | 82.29±0.33 | 85.35±0.09 |
| EVA-02 ViT-B/16 | 49.58±1.03 | 53.00±0.89 | 53.83±0.12 | 53.33±0.31 | 72.45±0.32 | 76.86±0.14 | 79.75±0.04 | 81.05±0.15 |
| DINO ViT-B/16 | 52.08±0.12 | 55.92±1.36 | 58.42±0.31 | 62.58±1.90 | 71.99±0.63 | 77.18±0.69 | 82.17±0.13 | 85.57±0.57 |
| SAM ViT-B/16 | 43.50±0.00 | 43.50±0.00 | 43.50±0.00 | 43.50±0.00 | 56.00±3.62 | 66.13±1.83 | 65.07±0.27 | 63.22±0.33 |
| $k$-NN ($k = 11$) | | | | | | | | |
| VGG16 | 47.75 | 51.25 | 53.25 | 55.75 | - | - | - | - |
| AlexNet | 46.75 | 48.25 | 52.75 | 54.75 | - | - | - | - |
| ResNet-18 | 47.50 | 49.00 | 51.00 | 53.50 | - | - | - | - |
| DenseNet-121 | 49.50 | 48.75 | **55.00** | 58.00 | - | - | - | - |
| EfficientNet-B4 | 49.75 | 52.00 | 54.75 | 51.00 | - | - | - | - |
| ViT-B/16 | 48.50 | 48.75 | 50.25 | 56.25 | - | - | - | - |
| CLIP ViT-B/16 | **52.25** | 48.75 | 50.00 | 52.75 | - | - | - | - |
| EVA-02 ViT-B/16 | 51.75 | 49.75 | 50.75 | 54.50 | - | - | - | - |
| DINO ViT-B/16 | 48.00 | **52.50** | 51.00 | **59.00** | - | - | - | - |
| SAM ViT-B/16 | 49.25 | 52.00 | 49.75 | 52.25 | - | - | - | - |

**Supplementary Table S12.** Benchmark outcomes summarizing the mean and standard deviation of accuracy (ACC), for a fixed operating point of 0.5, and area under the receiver operating characteristic curve (AUC) for the TissueMNIST dataset across all training scheme-model-image resolution combinations, derived from three independent random seeds. Notably, the $k$-NN algorithm, devoid of a training phase, remains unaffected by the stochasticity inherent in model training, thus reporting only the total ACC value without standard deviation. Moreover, owing to its direct utilization of embeddings and labels for classification, $k$-NN does not furnish a reliable AUC score. The overall best result across all training schemes, models, and resolutions is highlighted with a background color; the best result per resolution across all training schemes and models is highlighted with underline; and the best result per training scheme and resolution is highlighted in **bold**.

| TissueMNIST | | | | | | | | |
|---|---|---|---|---|---|---|---|---|
| Methods | Accuracy (ACC) | | | | Area Under the ROC Curve (AUC) | | | |
| | 28 × 28 | 64 × 64 | 128 × 128 | 224 × 224 | 28 × 28 | 64 × 64 | 128 × 128 | 224 × 224 |
| END-TO-END | | | | | | | | |
| VGG16 | **67.75±0.46** | 71.29±0.81 | 71.62±0.16 | 70.57±0.35 | **92.67±0.12** | 94.12±0.23 | 94.37±0.10 | 94.05±0.06 |
| AlexNet | 59.80±0.43 | 64.12±0.25 | 67.06±0.06 | 69.25±0.33 | 88.79±0.11 | 91.16±0.04 | 92.56±0.07 | 93.50±0.08 |
| ResNet-18 | 63.02±0.10 | 67.36±0.20 | 70.17±0.73 | 69.35±0.67 | 90.59±0.08 | 92.65±0.09 | 93.65±0.21 | 93.57±0.13 |
| DenseNet-121 | 66.53±0.37 | **71.54±0.71** | **74.25±0.39** | **74.08±0.32** | 92.47±0.04 | **94.46±0.24** | **95.35±0.07** | **95.25±0.04** |
| EfficientNet-B4 | 59.92±0.55 | 65.16±1.63 | 71.35±0.24 | 69.31±1.50 | 88.97±0.50 | 91.59±0.71 | 94.15±0.06 | 93.35±0.53 |
| ViT-B/16 | 60.55±0.56 | 66.72±0.71 | 71.29±0.40 | 72.89±0.70 | 88.97±0.18 | 92.62±0.31 | 94.32±0.17 | 94.84±0.16 |
| CLIP ViT-B/16 | 56.63±0.53 | 62.97±0.67 | 66.47±0.29 | 66.25±0.28 | 86.44±0.27 | 90.55±0.35 | 92.32±0.25 | 92.20±0.11 |
| EVA-02 ViT-B/16 | 57.60±1.00 | 64.42±0.67 | 70.42±0.87 | 70.34±0.93 | 87.31±0.58 | 91.21±0.29 | 93.92±0.33 | 93.97±0.35 |
| DINO ViT-B/16 | 59.44±0.31 | 65.80±1.01 | 69.35±1.72 | 70.38±0.99 | 88.55±0.27 | 91.98±0.44 | 93.48±0.77 | 94.02±0.37 |
| SAM ViT-B/16 | 58.86±0.20 | 66.19±0.70 | 69.42±0.60 | 71.47±0.41 | 88.07±0.32 | 92.25±0.33 | 93.70±0.23 | 94.40±0.13 |
| LINEAR PROBING | | | | | | | | |
| VGG16 | 53.19±0.00 | 53.53±0.02 | 55.50±0.08 | 58.12±0.13 | 83.95±0.00 | 84.03±0.00 | 86.36±0.03 | 87.89±0.04 |
| AlexNet | 49.10±0.10 | 53.80±0.06 | 55.75±0.06 | 59.57±0.05 | 80.35±0.13 | 84.74±0.06 | 86.47±0.03 | 88.98±0.01 |
| ResNet-18 | 51.06±0.00 | 53.43±0.02 | 54.60±0.02 | 56.46±0.01 | 82.44±0.00 | 84.76±0.00 | 85.65±0.00 | 86.97±0.00 |
| DenseNet-121 | 55.93±0.01 | 59.46±0.02 | 60.86±0.02 | 61.09±0.02 | 86.45±0.00 | 88.64±0.00 | 89.38±0.01 | 89.64±0.01 |
| EfficientNet-B4 | 54.24±0.01 | 56.91±0.00 | 57.88±0.02 | 58.47±0.01 | 85.29±0.00 | 87.17±0.00 | 87.63±0.00 | 88.33±0.00 |
| ViT-B/16 | 53.91±0.06 | 60.69±0.09 | 62.79±0.02 | 63.80±0.08 | 84.95±0.03 | 89.27±0.03 | 90.39±0.01 | 90.88±0.06 |
| CLIP ViT-B/16 | 55.28±0.03 | 59.39±0.04 | 61.03±0.08 | 61.50±0.06 | 86.02±0.01 | 88.60±0.02 | 89.62±0.01 | 89.76±0.08 |
| EVA-02 ViT-B/16 | 54.23±0.01 | 58.35±0.02 | 59.71±0.00 | 60.51±0.02 | 85.14±0.00 | 87.93±0.00 | 88.68±0.00 | 89.24±0.00 |
| DINO ViT-B/16 | **57.46±0.22** | **63.09±0.02** | **63.92±0.05** | **64.04±0.12** | **87.22±0.10** | **90.51±0.02** | **91.02±0.01** | **90.98±0.03** |
| SAM ViT-B/16 | 37.70±0.00 | 41.70±0.00 | 46.05±0.01 | 46.66±0.01 | 68.59±0.01 | 73.73±0.01 | 76.61±0.00 | 76.89±0.00 |
| $k$-NN ($k = 11$) | | | | | | | | |
| VGG16 | 47.30 | 47.20 | 48.55 | 51.30 | - | - | - | - |
| AlexNet | 45.97 | 49.96 | 50.79 | 54.11 | - | - | - | - |
| ResNet-18 | 48.25 | 48.89 | 49.05 | 51.54 | - | - | - | - |
| DenseNet-121 | 48.66 | 50.10 | 51.04 | 52.67 | - | - | - | - |
| EfficientNet-B4 | 49.79 | 51.14 | 50.35 | 51.26 | - | - | - | - |
| ViT-B/16 | 47.47 | 50.73 | 52.84 | 54.33 | - | - | - | - |
| CLIP ViT-B/16 | 48.04 | 50.48 | 53.29 | 53.46 | - | - | - | - |
| EVA-02 ViT-B/16 | 49.74 | 50.92 | 52.52 | 53.86 | - | - | - | - |
| DINO ViT-B/16 | **51.56** | **56.10** | **57.39** | **57.12** | - | - | - | - |
| SAM ViT-B/16 | 48.00 | 49.67 | 49.64 | 46.97 | - | - | - | - |
